# The importance of the nasopharynx and anterior skull base in excerebration techniques from KV40, a New Kingdom Egyptian site

**DOI:** 10.1002/ar.24828

**Published:** 2021-11-27

**Authors:** Roger Seiler, Patrick Eppenberger, Susanne Bickel, Frank Rühli

**Affiliations:** ^1^ Institute of Evolutionary Medicine (IEM) University of Zurich Zurich; ^2^ Department of Ancient Civilisations University of Basel Basel Switzerland

**Keywords:** excerebration, Kings' Valley, KV40, nonroyal tomb, transethmoidal/transsphenoidal craniotomy

## Abstract

In ancient Egypt, a unique technique for removing the brain was invented as part of the mummification practice and refined over the centuries. This usually involved piercing the anterior skull base through a nasal passage to remove the brain remnants through that perforation. From 2010 to 2018, an interdisciplinary team of the Universities of Basel and Zurich investigated tomb no. 40 (KV40) in the Valley of the Kings, Luxor, Egypt. Archaeological findings indicate a first burial phase during the mid‐18th Dynasty (ca. 1400–1350 BCE) and a second in the 22nd to 25th Dynasty (approx. 900–700 BCE). Repeated looting since ancient times severely damaged and commingled the human remains of the two burial phases. The detailed examination of the skulls showed evidence of different transnasal craniotomy practices. This study aims to provide a systematic presentation of the evidence for different excerebration techniques found in the mummy heads, skulls, and skull fragments from KV40, reflecting the long period of occupancy of this tomb by individuals of different social classes.

**Abbreviations**
ASB: anterior skull base
BCE: before common era
KV40: tomb no. 40 in the Kings' Valley
MNI: minimal number of individuals
TNC: transnasal craniotomy

## 1 INTRODUCTION

Ancient Egyptian mummification spans nearly four millennia and has continuously evolved throughout its long history. Its ritual significance is preserving the body so that the soul can recognize it and reunite with it in the afterlife. The evisceration, that is, removing the viscera to prevent rapid decomposition, has been a central feature of Egyptian anthropogenic mummification since its beginnings in the early Old Kingdom. The evisceration peaked in the New Kingdom and the Third Intermediate Period by spreading outside the pharaonic groups and became accessible to the elite sphere (Wade & Nelson, 2013). The brain was not removed consistently at first. Beginning as a rare practice with a prevalence of a few percent in the Old Kingdom, it was performed in about half of the mummifications in the New Kingdom, and—after a slight decline during the Third Intermediate Period—in almost all mummifications in the Ptolemaic and Roman periods (Wade, 2012). Only a short note by Herodotus informs us about the removal of the brain as—possibly—part of the mummification process (*490/480 BCE—424 BCE, History, Book 2, 86): “The mode of embalming, according to the most perfect process, is the following: They take first a crooked piece of iron and with it draw out the brain through the nostrils, thus getting rid of a portion, while the skull is cleared of the rest by rinsing with drugs…” (Wade, 2012).

Also, during its long history, the technique of excerebration underwent several variations. With an almost vertical inclination of the chisel to the cranial base, the embalmers created a perforation through the anterior skull fossa in the ethmoidal bone area, where the thin lamina cribrosa could easily be penetrated. This localization is found in a case from the 11th Dynasty of the Middle Kingdom (Gupta et al., 2008). There is also a preference for this anterior transethmoidal route in the New Kingdom, when transnasal craniotomy (TNC) was increasingly practiced (Wade, 2012). If the chisel was inserted deeper into the nasal passage, the sphenoid bone was perforated, which is a more demanding technique than the perforation of the thin lamina cribrosa. However, this posterior transsphenoidal route was often less damaging to the face's outer structures (Marx & D'Auria, 1986), a criterion, which became more important with time. The posterior, transsphenoidal route was favored from the Third Intermediate Period and throughout the Late Period (Fanous & Couldwell, 2012). Embalming substances could be introduced into the empty skull vault through the artificially created entry, which pooled in the postcranium to form a fluid level. Therefore, even when the skull‐base is not (entirely) preserved, such a deposit of embalming substances in the rear part of the cranium indicates a performed excerebration. Moreover, not in all cases, the deceased's brains were removed but could also be left to spontaneous mummification. In such cases, remnants of the desiccated brain can often be detected in the skull, and the bony structures of the skull‐base are found intact.

Within the “University of Basel Kings' Valley Project,” an interdisciplinary team from Basel and Zurich, led by Prof. Susanne Bickel, Basel, and Prof. Frank Rühli, Zurich, respectively, examined tomb no. 40 (KV40) in the Kings' Valley near Luxor. Through a deep shaft and a corridor of about 6 m, the tomb opens to a central chamber (C) with dimensions of eight by 3 m. From there, three further chambers are accessible (E and F in the south, D in the north). The archaeological findings in KV40 indicate a first burial period during the mid‐18th Dynasty (ca. 1400–1350 BCE). Inscriptions on the pottery vessels allowed the identification of over 30 persons by name. By naming them as “King's Son” and “King's Daughter,” most of them can be identified as family members of Amenhotep III. However, attribution of these titles to specific mummies is not possible. During the 22nd to 25th Dynasty (approx. 900–700 BCE), the tomb was reused by the local priestly elite (Meyer, 2017). Repeated looting and fire damage left behind many fragmented mummified, partially mummified and skeletonized human remains, commingling the two burial periods. This study, therefore, aims to provide a systematic presentation of the evidence for different excerebration techniques found in the mummy heads, skulls, and skull fragments from KV40, reflecting the long period of occupancy of this tomb by individuals of different social classes.

## 2 MATERIALS AND METHODS

The skulls and skull fragments and the postcranial elements from Tomb KV40 were inventoried and examined using a transdisciplinary workflow established to study these highly fragmented and intermingled human remains (Meyer et al., 2020). The mummified and skeletal remains of skulls and mandibles were found in varying preservation states—from intact skulls with preserved mummified soft tissues to small mandibular fragments calcified by fire. Potential excerebration defects can only be found with an intact middle part of the skull base's anterior half. That includes the median structures of the anterior skull base (ASB), the posterior wall of the frontal sinus, the naso‐ethmoidal region's roof with the *Crista Galli* and the *Lamina cibrosa* up to the spheno‐ethmoidal suture, and behind that the sphenoid bone up to the spheno‐occipital synchondrosis (Quirk & Connor, 2020). Only if these structures were preserved could a TNC be detected or excluded (non‐TNC). Thus, those specimens with only the skull vault preserved, or parts of the facial skull or the skull‐base but not the anterior parts, were excluded. On the other hand, fragments of skulls containing accumulations of embalming substances in the rear part, introduced after removing the brain, were found. Such findings also suggest a performed excerebration, even without direct evidence of the skull base's perforation.

Hence, the following findings may be distinguished:

- no TNC identified: non‐TNC;
- transethmoidal TNC, that is, a perforation in the area of the ethmoidal bone;
- transsphenoidal TNC (two of them with embalming substance in the rear part of the skull), that is, a perforation in the area of the sphenoidal bone;
- combined transethmoidal‐transsphenoidal path: large defect affecting both bones, so that the intended direction cannot be determined;
- fluid level formation of the embalming substance in the rear part of a fragmented skull.

Because in all cases, these defects are circumscribed and located internally and protected at the skull base, a taphonomic cause is unlikely. The approach for diagnosing a TNC differed according to the state of preservation of the specimen. In some cases, the skull calotte is destroyed, and only the anterior part of the skull base with the facial skull is preserved (e.g., specimen KV40_021). This allows a visual inspection of the ethmoid and sphenoid bones to assess the size and location of defects in this region. When the skull is completely preserved, a defect can be seen through the nasal passage, especially if the endonasal structures have been removed by the TNC (e.g., specimen KV40_002). In such cases, we documented them photographically with a DSLR camera (Nikon D 810, zoom lens 28–200 mm, macro lens 105 mm). In cases where a direct inspection was not feasible, that is, due to preservation of the entire skull or facial soft tissues, radiographic imaging was used. Plain radiographs were acquired with portable digital equipment, an X‐ray generator Examion PX 60 HF, Examion GmbH (voltage range, 40–100 kV; exposure range, 0.4–100 mAs; weight, 14.6 kg), and a flat panel detector, Examion DR 1417–600 WL, Examion GmbH, Fellbach, Germany (scintillator, gadolinium oxysulfide; active area, 358 × 430 mm; pixel matrix, 3,072 × 2,560 pixels; pixel pitch, 140 μm; grayscale, 14 bit; weight, 3.1 kg), in lateral, anterior–posterior and semi‐axial projections (Seiler, Eppenberger, et al., 2018). The latter, in particular, can be very suitable for displaying defects of the skull‐base. However, due to the superposition of the multiple endonasal structures and the structures of the skull‐base, diagnosing defects of the ASB is often challenging.

## 3 RESULTS

**FIGURE 1:**
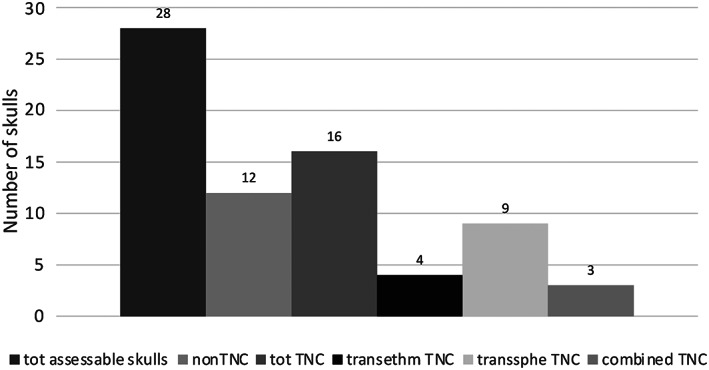
Number of found transnasal craniotomy (TNC) and non‐TNC cases in KV40

The state of preservation of the skulls is poor for all skeletal remains found in KV40 since plundering damages of the cranium are especially frequent (Salter‐Pedersen, 2004), and the fragile structures of the skulls are particularly prone to fracture. The minimal number of individuals (MNI), that, the smallest possible number of individuals found in KV40, is 83 (Meyer, 2017). Only 28 cases (33.7% of the skulls or parts thereof) were preserved to an extent, allowing a statement about a possible TNC. Twelve out of the 28 assessable skulls (42.8%) had no signs of a performed TNC (Tables 1 and 2, and Figure 1). The TNC cases group also includes five cases where only the posterior part of the skull calvaria was preserved where embalming substances had accumulated as a sign of a performed TNC. However, since no statement can be made about the perforation's localization and size, they were assigned to the non‐TNC category. Transsphenoidal TNC was carried out twice as much as transethmoidal TNC. Also, large defects of a combined transethmoidal–transsphenoidal route were found (“combined”).

**TABLE 1:** Number of found TNC and non‐TNC cases in KV40

Total assessable skulls	28	100%
Non‐TNC cases	12	42.86%
All TNC cases	16	57.14%

All TNC cases	16	100%
Cases with transethmoidal TNC	4	25.00%
Cases with transsphenoidal TNC	9	56.25%
Cases with combined TNC	3	18.75%

Abbreviation: KV40, tomb no. 40 in the Kings' Valley; TNC, transnasal craniotomy.

**TABLE 2:** The distribution of the found skulls of the individual categories in the various chambers of KV40

Chamber	C	D	E	F	Total
Non‐TNC	6	1	2	3	12
Transethmodial TNC	2	2	–	–	4
Transsphenoidal TNC	1	5	1	2	9
Combined TNC*	1	2	–	–	3
Embalming substance in skull	2	3	–	–	5
Total	12	12	3	5	33

Abbreviation: KV40, tomb no. 40 in the Kings' Valley; TNC, transnasal craniotomy.

*transethmoidal‐transsphenoidale route.

**FIGURE 2:**
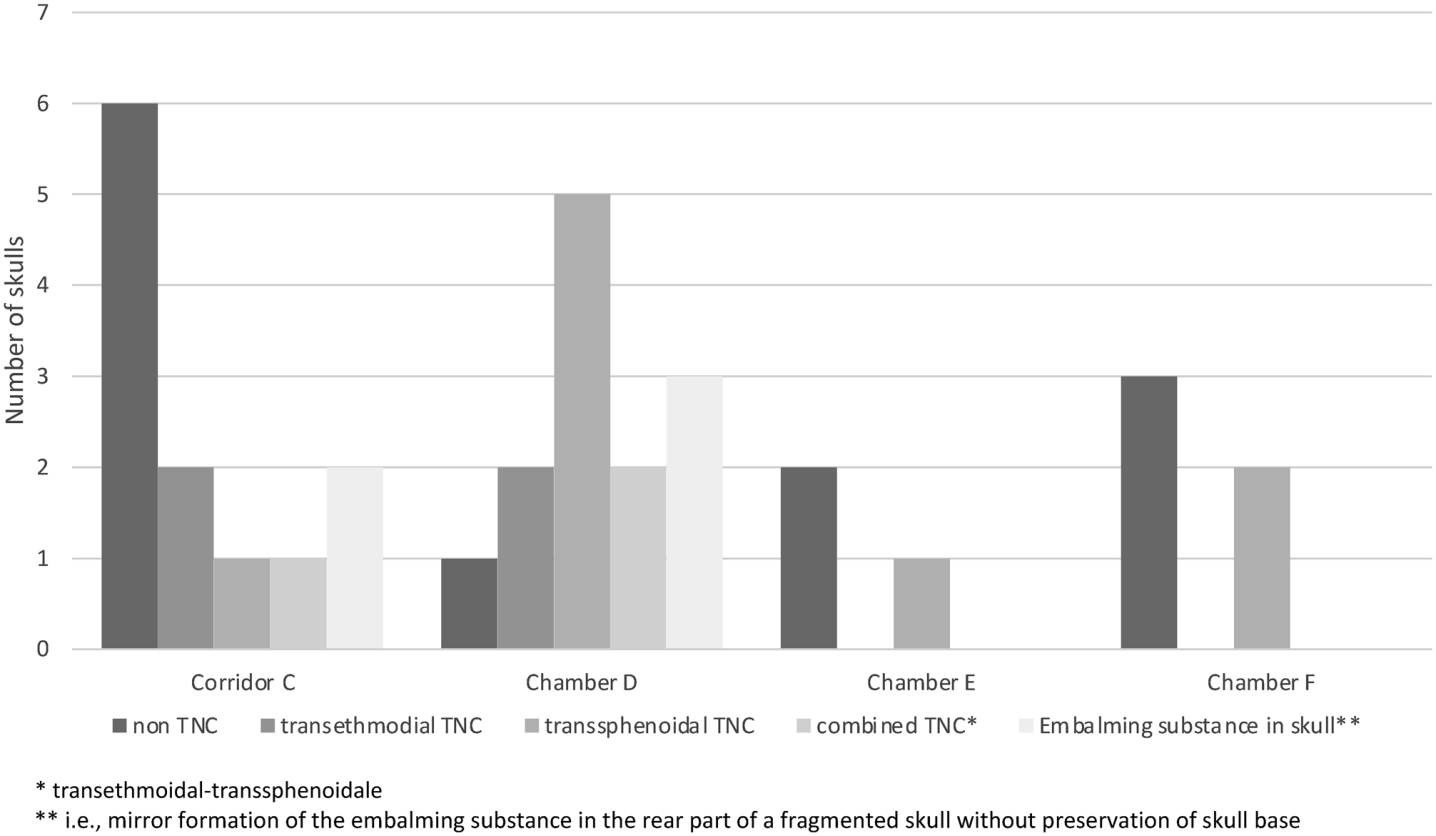
Distribution of transnasal craniotomy (TNC) categories in KV40

**FIGURE 3:**
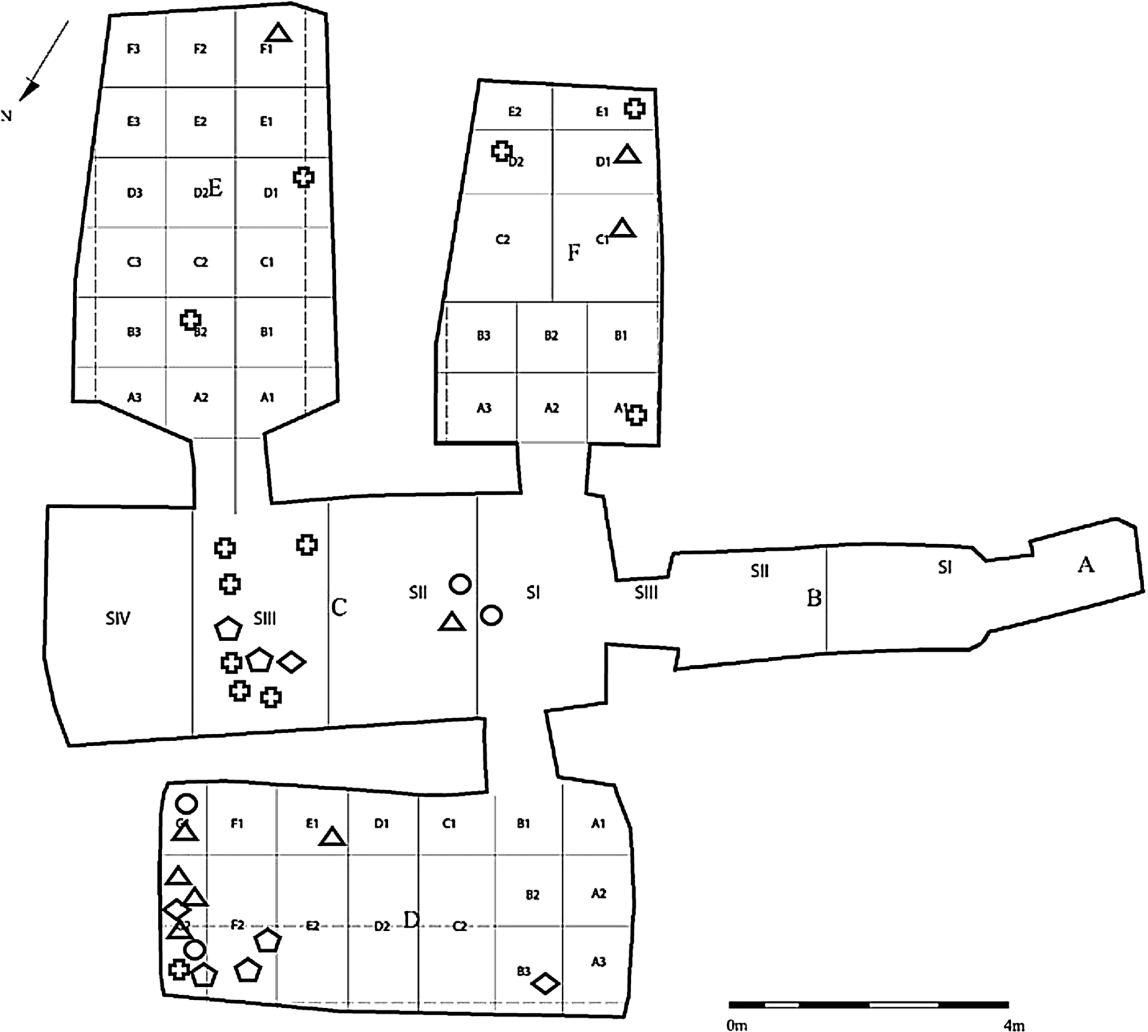
Plan of KV40 with approximate localization of the found skulls with and without TNC (circle: transethmoidal TNC; triangle: transsphenoidal TNC; diamond: combined TNC; cross: non‐TNC; pentagon: embalming substance in the skull). KV40, tomb no. 40 in the Kings' Valley; TNC, transnasal craniotomy

The non‐TNCs were found most frequently in central Chamber C, besides two cases found in Chamber E, three cases in Chamber F, and only one case in Chamber D. On the other hand, most transsphenoidal TNC cases were located in Chamber D and only exceptionally in the other chambers. The transethmoidal TNC cases were equally distributed between Chambers C and D (Figures 2 and 3). The soft tissues of the nose were preserved in only about one‐third of cases (6 of 16, 37.7%), including a nasal tampon in four cases (25.0%). In two cases with preserved noses, the bandages compressed the nasal soft tissues, and the instruments' access probably widened the left nostril, shifting the nose slightly to the contralateral side. Since the embalmers had to find their way to the skull base through the nasal passage with their instruments, this could result in damage or complete removal of the endonasal structures, with equal frequency regardless of the chosen excerebration route.

## 4 DISCUSSION

**FIGURE 4:**
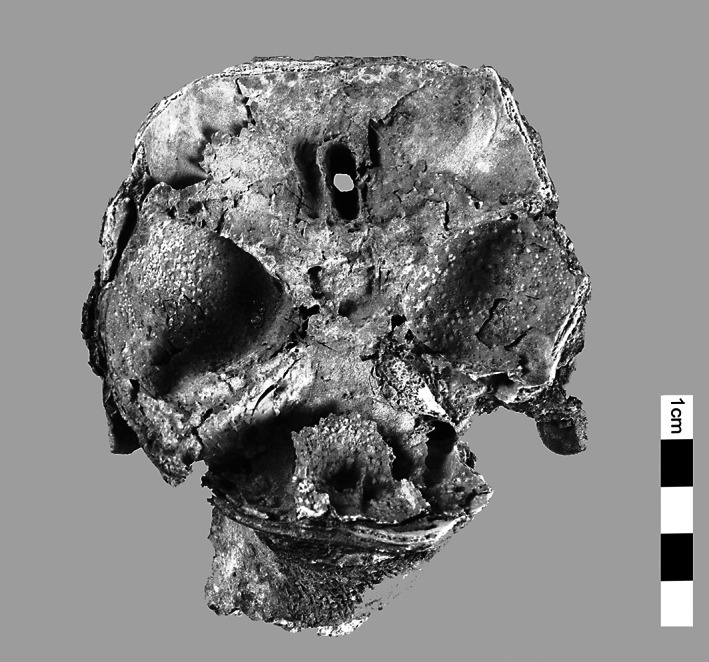
(KV40_025) Transethmoidal TNC in KV_40_025. KV40, tomb no. 40 in the Kings' Valley; TNC, transnasal craniotomy

A notable characteristic of KV40 are the numerous burials of children and even infants. Inscriptions on ceramic jars attest to several “royal daughters” or “royal sons.” Their age at death can, however, not be ascertained. The age of 12% of the individuals buried in KV40 was below three years (Meyer, 2017). These high‐ranking infant burials are characterized by elaborate and careful mummification, for the internal organs were mummified separately and placed back into the thoracic cavity as organ packages. The carefully performed transethmoidal craniotomy of an approximately 1‐month‐old infant KV_40_025 corresponds to such findings (Figure 4). Large parts of the cranium have broken away so that the skull base can be viewed directly. The lamina cribrosa is pierced by a longitudinal‐oval, cleanly delimited craniotomy on the crista Galli's right side. The nose and the anatomical structures in the nasal passage have been preserved.

**FIGURE 5:**
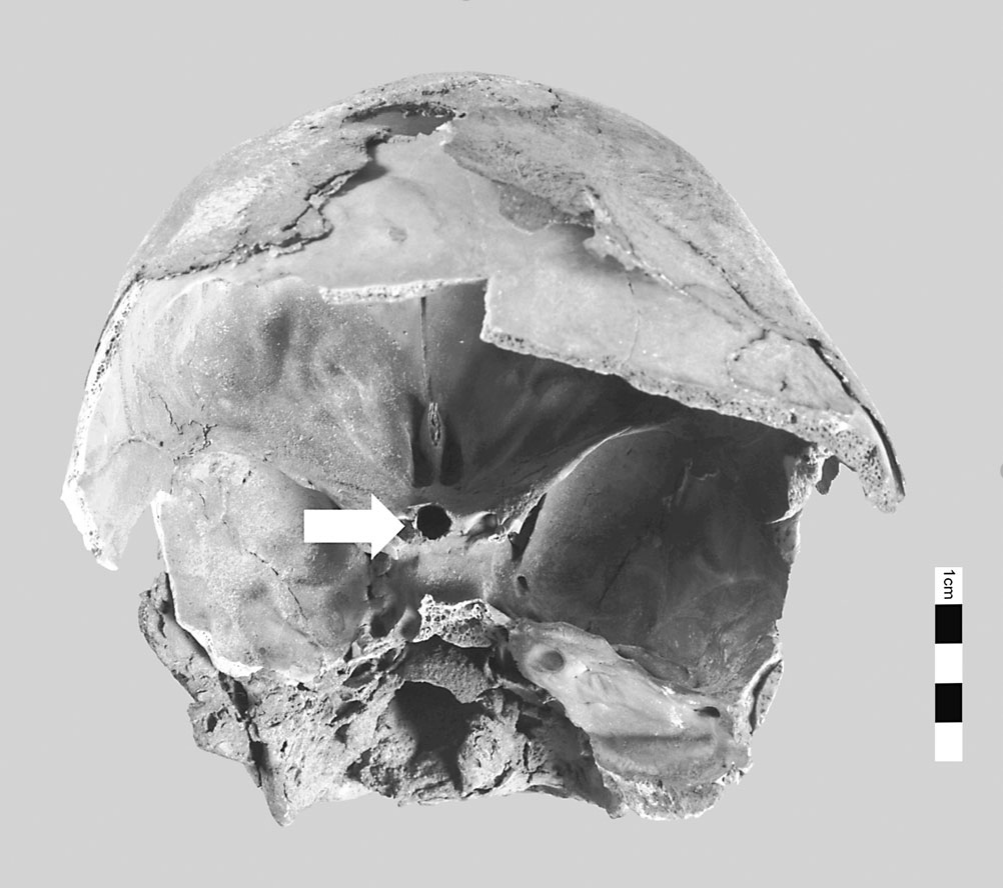
(KV40_51) Transspenoidal TNC (arrow). KV40, tomb no. 40 in the Kings' Valley; TNC, transnasal craniotomy

An even smaller perforation, measuring only about 12 mm in diameter, was found in an adolescent woman's sphenoid (KV_40_051). As the calvarium is missing, this TNC, limited to the sphenoid region, can be easily viewed directly (Figure 5). This contradicts the findings of Olszewski et al. (2020), who in their review found sphenoid fractures always to be associated with fractures of the ethmoid, that is, that the transsphenoidal TNC are only a posterior extension of the ethmoidal excerebration pathway. The almost circular defect, in this case, covers an area of about 113 mm^2^.

Also, the endonasal structures were preserved. The nose is obstructed with a bilateral nasal tamponade. How precisely this small defect was punched out becomes evident by comparison with other studies, where such defects were measured from Computed tomography (CT) data. The average defect size, measured in CT scans of the mummies from the Swiss Mummy Project (Rühli, 2016), a temporally and geographically inhomogeneous series of ancient Egyptian mummies from various Swiss museums, is 360 mm^2^ (Rentsch, 2018). In the mummy of Nakht‐ta‐Netjeret (ca. 950 BCE, Musée d'Ethnographie de Neuchâtel, Switzerland, inventory number: e.g., 185.c.), the brain was removed through a similarly small defect measuring 129 mm^2^, and notably, the mummies meninges, falx cerebri, and tentorium had been preserved (Seiler, Habicht, et al., 2018).

**FIGURE 6:**
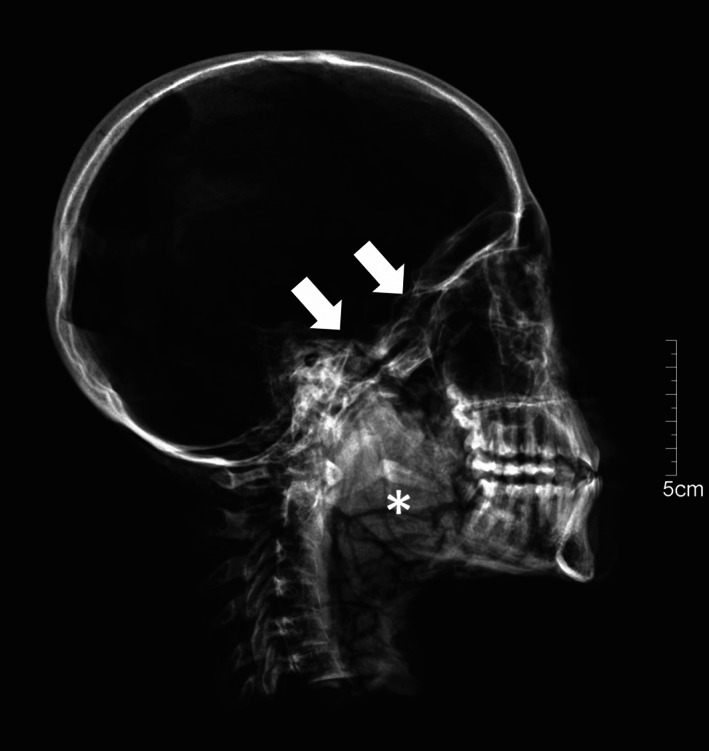
(KV40_026 X‐ray) X‐ray, lateral projection, transsphenoidal TNC (arrows), dislocated tooth (asterisk). KV40, tomb no. 40 in the Kings' Valley; TNC, transnasal craniotomy

Such careful executions of the transethmoidal (KV_40_025) or transsphenoidal (KV_40_051) TNC are striking. Although a small defect dimension is indicative of a particularly careful and skillful excerebration, it still does not necessarily correlate with social class, as Elhadi confirms (Elhadi et al., 2012). Consequently, the CT scans of pharaoh Amenhotep III, from whose royal court several burials in KV40 came from, reveal a defect in the skull base of 34 mm by 44 mm covering 1,175 mm^2^ (Hawass & Saleem, 2016). Also, there is a displaced tooth in Amenhotep III's oropharynx, as is the case in the mummified head of KV_40_026 (Figure 6).

A large perforation was set, and the upper left central incisor was dislocated into the oropharynx – as seen in the semi‐axial anteroposterior skull projection and the lateral X‐ray. In both cases, intravital deposition of the tooth can be ruled out. The dislocation of the teeth in later times by careless handling is improbable. In the mummy head KV_40_026, the peroral soft tissues are preserved, and Amenhotep III's oral cavity and oropharynx were filled with resin during mummification (Hawass & Saleem, 2016). Instead, it can be assumed that the tooth was accidentally luxated during the mummification process, or more precisely during the mouth opening procedure (Seiler & Rühli, 2015)—not the mouth opening ritual during the burial ceremony.

**FIGURE 7:**
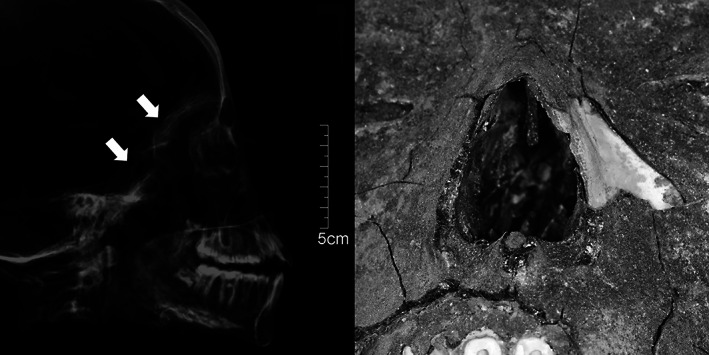
(KV40_024) Lateral X‐ray projection (left) and frontal view (right): destruction of endonasal structures and defects in the anterior skull base. KV40, tomb no. 40 in the Kings' Valley

Nearly half of the assessable cases in KV40 did not exhibit signs of an excerebration. As the frequency of TNCs is approximately the same in the New Kingdom and the late period (Wade et al., 2011), no difference between the two burial periods was expected for KV40, and the skulls cannot be assigned to the first or second burial phase based on this parameter. In contrast, as mentioned above, a temporal change in the choice of access to the skull interior is observed. In the New Kingdom, excerebration became common during mummification and was done initially through the transnasal–ethmoidal route (Fanous & Couldwell, 2012; Hoffmann & Hudgins, 2002; Oetteking, 1908; Pirsig & Parsche, 1991). Later this localization migrated posteriorly to reach the posterior, transsphenoidal route by the 25th and 26th Dynasties. Unfortunately, only a few skulls with transethmoidal perforations are preserved. They can probably be attributed to the first burial phase. However, the tomb suffered from the massive looting, which may have already occurred during the late 20th and 21st Dynasties. An exception may be the infant burials mentioned above, found only in Chambers C and D. They likely originate from the royal entourage during the first occupation phase. Due to the carefully performed transethmoidal excerebration and its location in Chamber D, infant skull KV_40_025 is also thought to belong to this first occupation phase. Transsphenoidal TNC was also found mainly in Chamber D along with transethmoidal–transsphenoidal TNC (Figure 7) and the resin containing calvaria, which likely belonged to the second occupation phase and accounted for almost one‐third of the burials. An almost similar number of burials are found in Chamber C but representing primarily non‐TNC cases, which cannot be attributed to any burial phase.

The distribution of individuals in the different chambers, as reported by Meyer (2017), corresponds to the distribution of the preserved skulls in those chambers. Therefore, Chambers C and D seem to have been the preferred burial locations during both occupation phases. It is unlikely that the bones of the early burials were systematically removed from where they were buried during the second burial phase or during looting (Meyer, 2017). So, despite many of the skull finds in KV40 being badly damaged and incomplete, they were most likely found in their approximate original deposition site. Unfortunately, however, there is no reliable dating for any of the mummies. Therefore, no skull can be attributed with certainty to one of the two occupation phases and thus also not to a specific social class. Such attributions have been attempted but must be considered hypothetical.

## 5 CONCLUSION

Artificial preservation of the deceased had been known in Europe since the Middle Ages, performed predominately for the funerals of royals, noblemen, or clerics: “…those who are by their dignity & their rank raised above the others would wish to get immortal.” (Penicher, 1699). After the evisceration—so Penicher—the surgeon “has to work at the head, from which he will saw the skull….” And finally, “he will join the separated bones of the skull, and the skin will be sewn back together.” Such an invasive and disfiguring procedure—hence the recommendation: surgeon “will cover the head with a hat…”—was unthinkable in Ancient Egypt. The deceased's bodies had to be preserved in their unaltered integrity through mummification, even including “restorations” of mutilated corpses by the embalmers (Giuffra et al., 2016; Gray, 1966; Ikram, 2015). In the Book of the Dead, the impairment of physical integrity was declared to be something terrible that, if it occurred, had to be overcome (Hermann, 1956). This was also true in particular for the face: “…here comes to you Hathor with beautiful face, the mistress of Dendara. She makes your face beautiful among the gods” (Töpfer, 2015, verse 4r; Macalister, 1894). The contour of the face, especially the nose, should be preserved in its shape: “…(for) the two nostrils two rolls of linen, ‘Protectionʼ is the name of the first, ‘Strengtheningʼ is the name of the other…” (Töpfer, 2015, verse 5). This required developing a technique to remove the brain without disfiguring the head's external structures and without breaking through the thick bone of the cranium. They entered through a sphenoethmoidal breach. Isolated but indisputable excerebrations can be found during the 12th Dynasty of the Middle Kingdom. The perforations in the ethmoid are large, and adjacent structures such as the orbit's median wall or even the palate were damaged. Above all, the embalmers also removed the nasal passage's anatomical structures for easy access (Strouhal, 1986). There was a long way to develop the surgical technique to a small, circular, sharply defined perforation, as seen in KV_40_051. As different as the techniques and their executions are, as different are the appraisals in the literature. For Harbort et al. (2008), a similar sizeable trans‐sphenoidal hole in a skull from the early Roman period (30 BC–395 ACE) is evidence for “a crude technique of brain removal.” Gaafar, on the other hand, sees “a well‐studied technique […] capable of creating a uniform clean‐cut endonasal craniotomy” (Gaafar et al., 1999) through the “more refined and esthetically pleasing transsphenoidal route” (Marx & D'Auria, 1986). KV40 offers a fascinating insight into the diversity of excerebration techniques of two distinct periods and two distinct populations of ancient Egypt—and how this challenging operation was well mastered—or sometimes not.

## AUTHOR CONTRIBUTIONS

**Roger Seiler:** Conceptualization (equal); data curation (lead); formal analysis (lead); investigation (lead); methodology (equal); validation (equal); visualization (equal); writing – original draft (lead). **Patrick Eppenberger:** Validation (equal); visualization (equal); writing – original draft (supporting); writing – review and editing (lead). **Susanne Bickel:** Funding acquisition (equal); investigation (equal); resources (supporting); supervision (lead). **Frank Rühli:** Conceptualization (equal); funding acquisition (equal); investigation (supporting); project administration (lead); resources (lead); supervision (supporting).
